# Fatty acid metabolism in lambs supplemented with different condensed and hydrolysable tannin extracts

**DOI:** 10.1371/journal.pone.0258265

**Published:** 2021-10-06

**Authors:** Bernardo Valenti, Luca Campidonico, Antonio Natalello, Massimiliano Lanza, Saheed A. Salami, Alessandro Priolo, Andrea Serra, Mariano Pauselli, Giuseppe Luciano

**Affiliations:** 1 Dipartimento di Scienze Agrarie, Alimentari e Ambientali (DSA3) di University of Perugia, Perugia, Italy; 2 Dipartimento di Agricoltura, Alimentazione e Ambiente (Di3A), University of Catania, Catania, Italy; 3 School of Food and Nutritional Sciences, College of Science, Engineering and Food Science, University College Cork, Cork, Ireland; 4 Dipartimento di Scienze Agrarie, Alimentari e Agro-ambientali, University of Pisa, Pisa, Italy; University of Illinois, UNITED STATES

## Abstract

Five groups of lambs (n = 9 each) were used to test the effect of plant extracts rich in hydrolysable (HT) or condensed tannin (CT) on animal performance, fatty acid composition of rumen content, liver and meat. The control group (CO) received a concentrate-based diet without tannins supplementation. The other groups received the same diet as the control lambs plus 4% chestnut (CH) and tara (TA) extracts as a source of HT and mimosa (MI) and gambier (GA) extracts as a source of CT. One-way ANOVA was used to assess the overall effect of dietary treatments, tannins supplementation (CO vs. CH+TA+MI+GA) and the effect of tannin type (HT vs. CT: CH+TA vs. MI+GA) on animal performance, rumen content, liver and intramuscular FA. Dietary CH negatively affected animal performance. The rumen content of the different groups showed similar levels of 18:3 *c*9*c*12*c*15, 18:2 *c*9*c*12, 18:2 *c*9*t*11, 18:1 *t*11 and 18:0, whereas 18:1 *t*10 was greater in CO. Also, 18:1 *t*10 tended to be lower in the rumen of HT than CT-fed lambs. These data were partially confirmed in liver and meat, where CO showed a greater percentage of individual *trans* 18:1 fatty acids in comparison with tannins-fed groups. Our findings challenge some accepted generalizations on the use of tannins in ruminant diets as they were ineffective to favour the accumulation of dietary PUFA or healthy fatty acids of biohydrogenation origin in the rumen content and lamb meat, but suggest a generalized influence on BH rather than on specific steps.

## Introduction

Tannins are water-soluble phenolic compounds that are produced by plants in response to external stressor factors [1], Chemically, tannins can be broadly classified into hydrolysable (HT) and condensed (CT). Hydrolysable tannins are polymers of gallic or ellagic acid associated by the mean of an ester bond with a polyhydroxylated core, whereas CT are represented by flavan-3-ol polymers [2]. The progressive interest of the researchers on the inclusion of tannins in the diets for ruminants is related to the capability of tannins to create stable complexes with carbohydrates, protein and ions, thus affecting the availability of nutrients [3]. Moreover, they can interact with the bacterial metabolism resulting in antimicrobial activity that may ultimately affect the composition and the functional activity of the rumen microbial community [4, 5]. The administration of dietary tannins has often led to contradictory results, which does not allow the full exploitation of their potential benefits. An early study described tannins as an anti-nutritional factor in ruminant diets [6], while, more recently, the administration of limited doses of tannins has been reported to improve the protein utilization along the digestive tract, as well as the growth and animal health [7]. Similarly, dietary tannins could be implemented as a strategy to manipulate the fatty acid composition of ruminant products by modulating the rumen biohydrogenation (BH), by which ruminal microflora progressively saturate the unsaturated fatty acids ingested with the diet. The effects of different tannins on ruminal BH are still unclear though it represents one of the most investigated subjects related with the inclusion of tannins in ruminant diets. Recently, Frutos et al. [8] reviewed the effect of tanniferous sources on a broad range of fatty acids in ruminant meat and milk because of their modulatory effect on ruminal microflora and lipid metabolism. In particular, *in vitro* [9, 10] and *in vivo* [11, 12] trials seem to suggest that tannins can interfere with each of the several steps along the BH process, thus determining the accumulation of different intermediate products. However, such effects are considerably variable.

The variable effect of tannins could be due to several factors. In the rumen environment, tannins can be differently metabolized and the capacity to interact with feeds, bacteria and microbial molecules may depend not only on their concentration in the diet but also on their chemical characteristics [13], such as the type of monomers linked to the sugar core or the polymerization degree. Differences in the chemistry of dietary tannins could, therefore, exert different effects on ruminant performance and product quality. Also, the basal diet could represent a confounding factor because tannins activity may differ, for example, when a forage-based diet is compared with a concentrate-based diet [14], or when diets differing for the level and composition of polyunsaturated fatty acids are compared [15] Also, when tanniniferous plants are fed to animals, several factors affect the concentration and composition of tannins and plant factors other than tannins may also contribute to the animal’s response. In this context, the inclusion of purified tannin extracts in the ruminant diet could help to clarify the effects of tannins. So far, most of the in vivo studies assessing the effect of dietary tannins on ruminant meat and milk fatty acids focused on one specific extract [14, 16], conversely in a limited number of studies the effect of different extracts has been investigated [17, 18]. Additionally, despite the market availability of several plant extracts providing different kind of tannins, quebracho (*Schinopsis lorentzii*) and/or chestnut (*Castanea sativa*) were used in most of the studies to supplement ruminant diets with condensed and hydrolysable tannins, respectively. However, several other plant extracts are commercially available and are characterized by possessing specific and different chemical groups of tannins.

In a preceding paper [19], we characterised the ruminal fermentation and microbiome in lambs supplemented with four different tannin extracts rich in hydrolysable or condensed tannins and we found that in a long-term feeding trial the extracts did not compromise ruminal fermentation, but showed specific antimicrobial activity against rumen microflora, which may elicit differences on fatty acids metabolism and specifically on PUFA biohydrogenation. Therefore, in the present study, using the same animals of the preceding paper, we evaluated and compared the effect of the dietary administration of chestnut (*Castanea sativa*) and tara (*Cesalpinia spinosa*) extracts as a source of HT, mimosa (*Acacia mearnsii*) and gambier (*Uncaria gambir*) extracts as a source of CT on the animal performance and fatty acid composition of rumen content, liver and meat in lambs, which represent three important districts implicated in the fatty acid metabolism.

## Materials and methods

### Animals and diets

This study was carried out indoors in the facilities of the University of Catania. This study was approved by the national veterinary authority. According to the Italian regulation (D. Lgs. n. 26/2014, art. 2, point 1 letter a and f) transposing the European Union Directive No.63/2010, the rules on the protection of animals used for scientific purposes do not apply to this study and an approval of the experimental plan from the ethical committee was not required. Nevertheless, the whole trial was performed under the supervision of the local veterinary authority and animals were handled in accordance with the European Union Directive No. 63/2010 on the protection of animals used for scientific purposes. Full details on the experimental plan are reported by Salami et al. [19]. In brief, forty-five 2-month-old male Sarda × Comisana lambs (initial body weight (BW) 19.6 ± S.D. 2.06 kg) were reared in individual pens where fresh water was always available for the duration of the trial. Lambs were equally divided into 5 groups (n = 9) that were balanced for the initial BW. One animal from the control group died during the adaptation period due to reasons not linked with the study. The control group (CO) exclusively received a barley-based concentrate diet containing on an “as fed” basis: barley (480 g/kg), wheat bran (230 g/kg), dehydrated alfalfa hay (150 g/kg), soybean meal (100 g/kg), molasses (20 g/kg) and mineral-vitamin premix (20 g/kg). The diet was formulated in order to provided about 2500 kcal/d of metabolizable energy and the SRNS system (ver. 1.11.7154.28131) was used. The four experimental groups received the same diet as the control lambs with the addition of 4% (as fed) of chestnut (CH) and tara (TA) extracts as a source of hydrolysable tannins and mimosa (MI) and gambier (GA) extracts as a source of condensed tannins. Chestnut, tara and mimosa extracts were purchased from Silvateam S.p.A. (San Michele M.vì, Cuneo, Italy), while gambier extract was purchased from Figli di Guido Lapi S.p.A. (Castelfranco di Sotto, Pisa, Italy). All the concentrates were in the form of a pellet and the tannins extracts were added before pelleting at the temperature of 40°C. Table 1 reports the chemical and fatty acid composition of the experimental basal diet. The experiment lasted a total of 84 days. During a 9-day-adaptation period, the animals received the same commercial starter concentrate used for weaning, with gradual increases in the proportion of the experimental concentrates. During the experimental period (75 days), the lambs were fed *ad libitum* with their respective diets. Daily, before morning feeding, individual intakes were measured according to the refusal left by each animal. Weekly, lambs were weighed before morning feeding and samples of each experimental diets were collected. Feed efficiency was calculated as the ratio between DMI and ADG during the 75-day-experimental period. At the end of the experimental period, all the lambs were slaughtered on the same day at a commercial abattoir (CE.MA. S.R.L., Acireale, Italy) according to the European Union welfare guidelines. All the animals had free access to their respective diet and water until approximately 3 hours before slaughter. Lambs were first stunned by a captive bolt and then exsanguinated. Each carcass was immediately weighted to record the hot carcass weight.

**Table 1:** Ingredients and composition of the basal diet given to the lambs.

**Chemical composition (DM%)**	
** Dry matter**	89.7
** Crude Protein**	15.7
** Ether Extract**	2.59
** NDF**	30.4
** ADF**	16.0
** ADL**	3.62
** Ash**	7.01
**Fatty acids (g/100g total fatty acids)**	
** 14:0**	0.15
** 16:0**	19.2
** 18:0**	2.00
** 18:1 c9**	17.0
** 18:2 c9c12**	53.7
** 18:3 c9c12c15**	5.53

DM: Dry Matter; NDF: Neutral Detergent Fibre; ADF: Acid Detergent Fibre; ADL: Neutral Detergent Lignin.

### Feed sampling and analyses

Feed samples were collected three times during the trial, packed under the vacuum and stored at -30°C until analyses. Feed sample for analyses was obtained by mixing an equal amount of the collected subsamples. Neutral detergent fibre (NDF) was determined according to Van Soest et al. [20]. Furthermore, crude protein, crude fat (ether extract) and ash were analysed according to AOAC [21]. Lipid was extracted and converted to fatty acid methyl esters (FAME) with a 1-step procedure reported by Valenti et al. [22]. Gas chromatographic analysis was carried out as later described for meat. Fatty acids in feed were expressed as g/100g fatty acids.

### Rumen samples collection and analyses

Individual ruminal content was collected within 15 min from slaughter. The ruminal wall was cut with a scalpel and the whole rumen content was placed into a 4-L-plastic beaker. After homogenization with a ladle, the ruminal pH was measured using a pH meter (HI-110; Hanna Instruments, Padova, Italy). An aliquot of approximately 120 mL of rumen content was immediately placed in dry ice prior to storage at −80°C. Freeze-dried rumen content was trans-esterified as described by Natalello et al. [11].

The FAME profile was obtained by gas-chromatography as later described for the meat samples. Fatty acids were expressed as g/100g of total fatty acids. The biohydrogenation indexes (%) for 18:2c9c12, 18:3c9c12c15, and the biohydrogenation completeness (%) in rumen content were estimated as reported by Alves et al. [23]. Specifically, the biohydrogenation indexes (%) were calculated as follow:

$${BH}_{FAx}(\%)=\frac{\left({FAx}_{diet}-{FAx}_{rumen}\right)}{{FAx}_{diet}}\times 100$$

where:

BH_FAx_ (%): is the estimate proportion of FAx (18:2c9c12 or 18:3c9c12c15) that disappears between the diet and the rumen digesta; FAx_diet_: is the proportion of FAx in the diet, expressed as percentage of total C18-carbon FA; FAx_rumen_: is the proportion of FAx in the rumen, expressed as percentage of total C18-carbon FA.

The biohydrogenation completeness (%) was estimated as follow:

$$BH\;completeness\;(\%)=\frac{{C18:0}_{rumen}}{\begin{array}{c} {C18:0}_{diet}+(C18:1{c9}_{diet}-C18:1{c9}_{rumen})\\ +(C18:2{c9c12}_{diet}-C18:2{c9c12}_{rumen})\\ +(C18:3{c9c12c15}_{diet}-C18:3{c9c12c15}_{rumen}) \end{array}}\times 100$$

where:

BH completeness (%): is the estimation of the extent of the biohydrogenation of C18:1c9, C18:2*c*9*c*12 and C18:3*c*9*c*12*c*15 present in the diet; C18:0_diet_: is the C18:0 in the diet as percentage of total C18-carbon FA; C18:1*c*9_diet_: is the C18:1*c*9 in the diet as percentage of total C18-carbon FA; C18:1*c*9_rumen_: is the C18:1*c*9 in the rumen digesta as percentage of total C18-carbon FA; C18:2*c*9*c*12_diet_: is the C18:2*c*9*c*12 in the diet as percentage of total C18-carbon FA; C18:2*c*9*c*12_rumen_: is the C18:2*c*9*c*12 in the rumen digesta as percentage of total C18-carbon FA; C18:3*c*9*c*12*c*15_diet_: is the C18:3*c*9*c*12*c*15 in the diet as percentage of total C18-carbon FA; C18:3*c*9*c*12*c*15_rumen_: is the C18:3*c*9*c*12*c*15 in the rumen digesta as percentage of total C18-carbon FA.

### Liver and meat sampling and analysis

Immediately after slaughter liver was excised, vacuum packed and stored at -80°C pending analysis. After 24-hours storage at 4°C the carcass was halved and the pH measured (HI-110; Hanna Instruments, Padova, Italy) on the *longissimus thoracis et lumborum* (LTL) muscle on right side of the carcass. Thereafter, LTL was removed, packed under vacuum and stored at -80°C until analysis. Liver and intramuscular fat was extracted in double from 10 g of finely minced LTL samples with a mixture of chloroform and methanol (2:1, v/v) as described by Folch et al. [24] and 30 mg of lipids were converted to FAME by base-catalyzed transesterification [25], using 0.5 mL of sodium methoxide in methanol 0.5 N and 1 mL of hexane. Nonadecanoic acid was used as an internal standard. Gas chromatographic analysis was carried out with a GC 8000 Top ThermoQuest (Milan, Italy) equipped with a flame ionization detector (FID; ThermoQuest, Milan, Italy) and 100-m high-polar fused silica capillary column (WCOT-fused silica CP-Select CB for FAME Varian, Middelburg, the Netherlands; 100m×0.25mm i.d.; film thickness 0.25 μm). Helium was the carrier gas at a constant flow of 1 mL/min. Total FAME profile in a 1 μL sample volume (2 μL for the feeds and rumen lipid) at a split ratio of 1:80 was determined using the GC conditions reported by Valenti et al. [26]: the oven temperature was programmed at 40°C and held for 4 min, then increased to 120°C at 10°C/min, held for 1 min, then increased up to 180°C at 5°C/min, held for 18 min, then increased up to 200°C at 2°C/min, held for 15 min, and then increased up to 230°C at 2°C/min, held for 19 min. The injector and detector temperatures were at 270°C and 300°C, respectively. FAME identification was based on a standard mixture of 52 Component FAME Mix (Nu-Chek Prep Inc., Elysian, MN, USA) and individual FAME standards (Larodan Fine Chemicals, Malmo, Sweden) and published chromatograms [27, 28]. Fatty acids were expressed as g /100g of total fatty acids.

### Statistical analysis

As in the companion paper [19], one-way ANOVA was used to assess the overall effect of dietary treatments, tannins supplementation (control vs. CH+TA+MI+GA) and the effect of tannin type (HT vs. CT: CH+TA vs. MI+GA) on animal performance, rumen content, liver and intramuscular FA composition (Minitab version 16, Minitab Inc, State College, PA). The differences between means were separated using the Tukey’s adjustment for pairwise comparisons. Differences were declared significant at *P* ≤ 0.05. Furthermore, the FA composition of rumen content, liver and intramuscular fat were subjected to a multivariate linear discriminant analysis (SPSS, ver. 18.0.0, 2009), using a stepwise selection of those variables (FA) that better discriminated between the experimental groups, following the procedure described by Valenti et al. [29]. Variables with F < 0.10 were retained at the end of the stepwise procedure and submitted to canonical discriminant analysis. This procedure allows the creation of a linear function (CAN) that includes the quantitative variables that best summarize the variance of the dataset and maximize the distance between the groups. The discriminant capacity was evaluated through the Wilks’s lambda test of significance and the discriminating efficacy of the model was expressed as the percent correct assignment of each individual to the respective group using the *leave-one-out* cross-validation. In this procedure, each lamb is treated as an unknown subject and the canonical functions, previously developed with the entire dataset, are used to assign it to a group. The higher the discriminating capacity of the model, the higher the percentage of cases correctly assigned to their respective group.

## Results

### Animal performance

Table 2 reports data on the main animal performance parameters. The supplementation of tannins in the diet of lambs did not affect animal performance (*P* > 0.05), except for dry matter intake (DMI; *P* = 0.048), which was greater in the CO group than CH+TA+GA+MI. The multiple comparisons between treatment groups showed a significant effect of the diet on the DMI (*P* < 0.001), final body weight (BW, *P* = 0.009), average daily gain (ADG, *P* = 0.002), carcass weight (*P* = 0.004) and FE (*P* = 0.041). These results were principally due to the lower values observed in CH in comparison with the CO and the other groups. In particular, CH lambs showed a lower DMI than the CO group and the lambs fed condensed tannins, the latter explaining the significant effect of tannin type (HT *vs* CT) on this parameter (*P* = 0.001). The lower DMI in the CH group resulted in a lower final BW, ADG and carcass weight in comparison with CO, TA and GA. However, the FE resulted lower only in CH lambs than in the other group receiving hydrolysable tannins (TA).

**Table 2:** Effect of the different tannin extracts on feed intake, lamb performances and carcass characteristics.

	Dietary treatment^1^					SEM	*p* value	Contrast^2^	
	CO	CH	TA	GA	MI			Tannins	Tannin type
**DMI (kg/d)**	1.17^a^	0.92^b^	1.06^a.b^	1.09^a^	1.17^a^	0.028	<0.001	0.048	0.001
**Final BW (kg)**	35.5^a^	30.3^b^	35.8^a^	35.1^a^	34.8^a.b^	0.576	0.009	0.327	0.164
**ADG (kg/d)**	0.20^a^	0.13^b^	0.21^a^	0.20^a^	0.19^a^	0.007	0.002	0.397	0.098
**Feed efficiency**	5.34^a.b^	6.26^b^	4.69^a^	4.94^a.b^	5.47^a.b^	0.458	0.041	0.978	0.811
**Carcass weight (kg)**	17.1^a^	14.7^b^	17.8^a^	17.2^a^	16.7^a.b^	0.290	0.004	0.540	0.300
**Muscle pH**	5.91	5.93	5.89	5.90	5.98	0.019	0.582	0.769	0.467

DMI: Dry matter intake.

ADG: Average daily gain.

Feed efficiency calculated as the ratio between DMI and ADG during the 75-day-experimental period.

SEM: Standard error of mean.

^1^Dietary treatments: CO: Control; CH: Chestnut extract; TA: Tara extract; GA: Gambier extract; MI: Mimosa extract.

^2^Contrasts: Tannins (overall effect of tannin: CON vs. CH + TA + GA + MI); Tannin type (effect of HT vs. CT: CH + TA vs. GA + MI).

Dietary treatment means within a row with different superscripts differ at a significance of P ≤ 0.05.

### Rumen content, liver and meat fatty acids

Table 3 reports data on individual FA in the rumen content. The dietary treatment, the tannin supplementation and the tannin type did not affect the sum of saturated fatty acids (SFA), PUFA, PUFA n-3, PUFA n-6, the ratio PUFA n-6 to n-3 nor the biohydrogenation indexes. Among the individual SFA, only 22:0 was affected by the tannin supplementation (*P* = 0.001) and differed between tannins type (*P* = 0.045). Specifically, the rumen content of tannin-fed lambs had a lower proportion of 22:0 in comparison to CO and, between the two tannin types, HT showed a greater proportion than CT. The multiple comparisons between dietary treatment groups showed that the sum of odd- and branched-chain fatty acids (OBCFA) was not affected by the diet. However, an effect of tannin supplementation was observed on individual OBCFA, with 13:0 (*P* = 0.051) and 13:0*iso* (*P* = 0.022) greater in the in tannins-fed lambs in comparison with CO, whereas an opposite result was found for 17:0*anteiso* (*P* = 0.013). Regarding tannin type, the proportion of 17:0 and 17:0*iso* was greater in the rumen content of lambs fed CT (*P* = 0.024 and *P* = 0.033, respectively). Dietary treatment (*P* = 0.029) and tannins supplementation (*P* = 0.003) affected total monounsaturated fatty acids (MUFA). These results were mainly related to the greater proportion of 18:1*t*6+*t*7+*t*8 and 18:1*t*10 found in the rumen content of CO lambs in comparison to the other groups. Among the groups receiving tannins, only GA showed levels of 18:1*t*6+*t*7+*t*8 comparable to CO. As for the individual PUFA, TA lambs showed a double proportion of 18:2*t*9*c*12 in comparison with all the other groups, which caused that HT fed lambs had a greater proportion of this fatty acid in comparison to CT (*P* = 0.020). Similarly, 18:2*c*9*c*12 tended to be greater in HT lambs than CT (*P* = 0.075).

**Table 3:** Effect of different tannin extracts on the fatty acid composition of rumen content (g/100g total fatty acids).

	Dietary treatment^1^					SEM	*p* value	Contrast^2^	
	CO	CH	TA	GA	MI			Tannins	Tannins type
**12:0**	0.406	0.521	0.532	0.510	0.433	0.026	0.467	0.176	0.355
**13:0**	0.188	0.253	0.287	0.319	0.294	0.020	0.303	0.051	0.424
**13:0*iso***	0.178	0.287	0.266	0.316	0.285	0.019	0.213	0.022	0.579
**14:0**	1.483	1.605	1.685	1.960	1.541	0.082	0.369	0.314	0.585
**14:1*c*9**	0.212	0.222	0.271	0.287	0.345	0.024	0.442	0.283	0.232
**14:0*iso***	0.181	0.159	0.209	0.182	0.187	0.012	0.805	0.926	0.987
**15:0**	0.961	0.967	0.964	1.279	0.936	0.045	0.064	0.522	0.170
**15:0*iso***	0.367	0.375	0.362	0.418	0.457	0.023	0.668	0.599	0.187
**15:0*anteiso***	1.427	1.555	1.587	1.753	1.649	0.073	0.735	0.274	0.462
**16:0**	14.49	15.16	13.53	14.75	14.48	0.207	0.137	0.982	0.565
**16:1*c*9**	0.469	0.269	0.396	0.269	0.382	0.051	0.705	0.295	0.950
**16:0*iso***	0.196	0.174	0.112	0.159	0.211	0.012	0.069	0.306	0.125
**17:0**	0.567	0.437	0.409	0.560	0.495	0.024	0.137	0.142	0.024
**17:0*iso***	0.232	0.171	0.156	0.243	0.223	0.015	0.238	0.378	0.033
**17:0*anteiso***	0.508	0.375	0.325	0.299	0.365	0.026	0.127	0.013	0.671
**18:0**	30.04	34.89	32.89	31.59	33.06	1.530	0.908	0.447	0.661
**18:1*t*5**	0.366	0.417	0.459	0.610	0.498	0.032	0.151	0.114	0.086
**18:1*t*6+*t*7+*t*8**	1.868^a^	0.940^b^	0.977^b^	1.437^a.b^	1.116^b^	0.094	0.006	0.001	0.076
**18:1*t*9**	1.180	1.057	1.040	1.372	1.167	0.050	0.211	0.871	0.045
**18:1*t*10**	10.49^a^	2.877^b^	3.191^b^	4.671^b^	4.106^b^	0.582	<0.001	<0.001	0.066
**18:1*t*11**	1.736	1.931	1.546	1.689	2.404	0.133	0.281	0.656	0.337
**18:1*c*6**	1.302	1.273	1.366	1.451	1.263	0.051	0.764	0.787	0.755
**18:1*c*9**	6.190	6.638	7.467	6.526	7.010	0.264	0.629	0.298	0.649
**18:1*c*11**	1.049	1.042	1.165	1.319	0.862	0.067	0.274	0.787	0.936
**18:1*c*12**	1.238	1.151	1.293	1.536	1.529	0.084	0.497	0.530	0.113
**18:1*c*13**	0.138	0.167	0.222	0.252	0.207	0.020	0.442	0.162	0.447
**18:2*t*8*c*13**	0.094	0.148	0.096	0.133	0.124	0.010	0.340	0.221	0.732
**18:2*t*9*c*12**	0.293^b^	0.366^b^	0.723^a^	0.343^b^	0.384^b^	0.035	<0.001	0.071	0.020
**18:2*t*9*c*13**	0.141	0.114	0.095	0.156	0.150	0.012	0.443	0.696	0.078
**18:2*t*10*c*12**	0.036	0.037	0.091	0.128	0.080	0.012	0.091	0.135	0.181
**18:2*c*9*t*12**	0.276	0.299	0.267	0.411	0.424	0.037	0.495	0.437	0.111
**18:2*c*9*t*11**	0.696	0.749	0.828	0.924	0.894	0.050	0.586	0.237	0.267
**18:2*c*9*c*12**	5.711	8.180	8.771	5.300	7.510	0.497	0.104	0.183	0.075
**18:3*c*6*c*9*c*12**	0.102	0.161	0.222	0.218	0.208	0.020	0.291	0.050	0.644
**18:3*c*9*c*12*c*15**	0.446	0.650	0.738	0.510	0.564	0.042	0.202	0.119	0.106
**20:0**	0.589	0.588	0.558	0.637	0.671	0.028	0.719	0.738	0.216
**20:1*t*11**	0.093	0.207	0.265	0.181	0.200	0.028	0.435	0.093	0.487
**20:1*c*11**	0.313	0.297	0.363	0.334	0.301	0.023	0.888	0.859	0.824
**20:4n-6**	0.124	0.156	0.182	0.251	0.169	0.018	0.248	0.163	0.322
**20:5n-3**	0.376	0.278	0.302	0.245	0.287	0.029	0.722	0.191	0.695
**21:0**	0.025	0.047	0.069	0.038	0.037	0.006	0.258	0.167	0.157
**22:0**	0.589^a^	0.350^a.b^	0.170^b^	0.108^b^	0.138^b^	0.049	0.008	0.001	0.045
**22:1*c*13**	0.212	0.175	0.271	0.164	0.234	0.028	0.753	0.995	0.724
**22:2n-6**	0.039	0.051	0.037	0.023	0.034	0.008	0.867	0.905	0.297
**22:5n-6**	0.140	0.224	0.154	0.196	0.180	0.026	0.871	0.473	0.898
**22:5n-3**	0.039	0.047	0.033	0.042	0.052	0.006	0.902	0.807	0.634
**22:6n-3**	0.130	0.092	0.149	0.205	0.148	0.018	0.401	0.706	0.199
**23:0**	0.535	0.202	0.291	0.133	0.374	0.048	0.081	0.021	0.946
**∑ SFA**	47.60	53.12	49.36	49.56	50.32	1.530	0.864	0.458	0.708
**∑ MUFA**	26.86^a^	18.66^b^	20.29^b^	22.10^a.b^	21.62^a.b^	0.838	0.029	0.003	0.127
**∑ PUFA**	8.642	11.55	12.69	9.080	11.21	0.585	0.144	0.100	0.144
**∑ OBCFA**	5.367	5.00	5.036	5.698	5.511	0.190	0.744	0.913	0.189
**∑ PUFA n-6**	6.116	8.770	9.367	5.980	8.10	0.498	0.099	0.135	0.081
**∑ PUFA n-3**	0.992	1.067	1.222	1.002	1.050	0.055	0.700	0.517	0.342
**PUFA n-6/n-3**	6.528	8.282	7.751	6.502	7.943	0.350	0.357	0.234	0.324
**BH Indexes (%)** ^**3**^									
**Completeness**	59.0	72.4	69.7	65.0	67.1	1.920	0.256	0.055	0.211
**18:2c9c12**	86.7	80.7	79.7	87.4	82.85	1.200	0.151	0.200	0.083
**18:3c9c12c15**	89.9	85.1	83.3	87.2	86.9	1.020	0.353	0.110	0.234

SFA: saturated fatty acids; MUFA: monounsaturated fatty acids; PUFA: polyunsaturated fatty acids; OBCFA: odd and branched chain fatty acids; SEM: Standard error of mean.

^1^Dietary treatments: CO: Control; CH: Chestnut extract; TA: Tara extract; GA: Gambier extract; MI: Mimosa extract.

^2^Contrasts: Tannins (overall effect of tannin: CON vs. CH + TA + GA + MI); Tannin type (effect of HT vs. CT: CH + TA vs. GA + MI).

Dietary treatment means within a row with different superscripts differ at a significance of P ≤ 0.05.

^3^BH indexes: indexes of biohydrogenation of dietary unsaturated fatty acids calculated as reported by Alves et al. [20].

Table 4 reports data on the FA composition of liver fat. Dietary treatment affected (*P* ≤ 0.05) several *trans* 18:1 isomers in liver fat. Specifically, 18:1*t*9 and 18:1*t*10 were greater in MI than CH liver. Moreover, 18:1*t*10 was found at greater percentage in the liver fat of CO in comparison with CH, TA and GA. Also, 18:1*t*10 was greater in MI than CH, whereas the percentage of 18:1*t*11 was greater in CO than GA liver. Overall, tannins supplementation reduced (*P* ≤ 0.05) the percentage of 18:1*t*9, 18:1*t*10 and 18:1*t*11 in comparison with CO. Between the different classes of tannins, 18:1*t*10 was found at lower percentage (*P* = 0.037) in the liver fat of HT- lambs than CT-supplemented lambs. Regarding PUFA in liver fat, dietary treatment affected 20:5n-3, which was greater (*P* = 0.042) for CO than MI. Tannin supplementation overall reduced (*P* ≤ 0.05) 18:2*c*9*t*11 and 18:2*t*9*c*12 as compared to CO.

**Table 4:** Effect of different tannin extracts on liver fatty acid composition (g/100g total fatty acids).

	Diet^1^					SEM	*p* value	Contrast^2^	
	CO	CH	TA	GA	MI			Tannins	Tannin type
**Fat (g/100 liver)**	3.60	3.96	3.76	3.82	3.77	0.049	0.235	0.065	0.436
**12:0**	0.049	0.074	0.056	0.064	0.057	0.004	0.321	0.173	0.639
**14:0**	0.725	1.169	0.817	0.868	0.730	0.058	0.083	0.259	0.157
**14:0*iso***	0.010	0.024	0.021	0.014	0.015	0.002	0.432	0.191	0.189
**14:1*c*9**	0.030	0.044	0.026	0.029	0.028	0.003	0.312	0.870	0.353
**15:0**	0.404	0.462	0.409	0.442	0.377	0.017	0.571	0.688	0.542
**15:0*iso***	0.075	0.093	0.088	0.069	0.068	0.006	0.486	0.740	0.096
**15:0*anteiso***	0.138	0.161	0.163	0.130	0.127	0.010	0.709	0.788	0.185
**16:0**	14.662	15.973	14.827	15.565	14.791	0.281	0.519	0.396	0.738
**16:0*iso***	0.404	0.462	0.409	0.442	0.377	0.006	0.567	0.906	0.258
**16:1*c*7**	0.487	0.565	0.501	0.600	0.474	0.019	0.148	0.336	0.932
***C*16:1*c*9**	0.991	1.031	0.943	1.100	0.952	0.029	0.416	0.839	0.565
**17:0**	1.657	1.541	1.541	1.762	1.444	0.050	0.292	0.513	0.585
**17:0*iso***	0.498	0.478	0.454	0.460	0.502	0.011	0.541	0.380	0.560
**17:0*anteiso***	0.496	0.515	0.472	0.550	0.476	0.015	0.416	0.839	0.565
**17:1*c*9**	0.576	0.523	0.484	0.625	0.494	0.021	0.171	0.421	0.254
**18:0**	20.351	20.182	21.601	20.467	20.538	0.322	0.670	0.684	0.600
**18:1*c*6**	0.149	0.214	0.218	0.193	0.120	0.019	0.421	0.458	0.195
**18:1*c*9**	16.761	18.366	15.629	18.288	15.758	0.422	0.082	0.823	0.980
**18:1*c*11**	1.859	1.623	1.632	1.946	1.585	0.056	0.150	0.269	0.284
**18:1*c*12**	0.508	0.486	0.438	0.478	0.500	0.019	0.803	0.502	0.533
**18:1*c*13**	0.094	0.063	0.076	0.076	0.062	0.006	0.465	0.107	0.972
**18:1*t*5**	0.021	0.006	0.017	0.024	0.014	0.003	0.192	0.407	0.234
**18:1*t*6+*t*7+*t*8**	0.353	0.266	0.336	0.315	0.318	0.011	0.110	0.113	0.521
**18:1*t*9**	0.396^a^	0.242^b^	0.324^a.b^	0.297^a.b^	0.342^a^	0.013	0.002	0.004	0.150
**18:1*t*10**	2.375^a^	0.842^c^	1.247^b.c^	1.073^b.c^	1.676^a.b^	0.108	<0.001	<0.001	0.037
**18:1*t*11**	1.028^a^	0.762^a.b^	0.766^a.b^	0.583^b^	0.869^a.b^	0.045	0.027	0.013	0.663
**18:2*c*9*c*12**	10.637	10.425	11.810	10.781	11.886	0.246	0.175	0.362	0.709
**18:2*c*9*t*11**	0.491	0.368	0.400	0.395	0.446	0.017	0.169	0.040	0.261
**18:2*t*8*c*13**	0.118	0.140	0.113	0.146	0.117	0.006	0.311	0.472	0.698
**18:2*t*9*c*12**	0.108	0.085	0.065	0.078	0.074	0.005	0.118	0.016	0.931
**18:2*t*9*c*13**	0.178	0.236	0.162	0.245	0.192	0.011	0.063	0.265	0.418
**18:3*c*6*c*9*c*12**	0.275	0.266	0.279	0.251	0.304	0.013	0.763	0.998	0.881
**18:3*c*9*c*12*c*15**	0.423	0.435	0.558	0.507	0.533	0.019	0.108	0.093	0.592
**20:0**	0.275	0.266	0.279	0.251	0.304	0.004	0.998	0.780	0.979
**20:1*c*11**	0.233	0.175	0.193	0.214	0.157	0.010	0.137	0.069	0.945
**20:2*c*11*c*14**	0.232	0.208	0.244	0.175	0.246	0.010	0.146	0.604	0.542
**20:3n-6**	0.872	0.719	0.860	0.723	0.665	0.033	0.172	0.124	0.201
**20:3n-3**	0.016	0.036	0.024	0.029	0.030	0.003	0.467	0.122	0.973
**20:4n-6**	9.567	9.034	9.683	8.818	10.289	0.255	0.391	0.869	0.755
**20:5n-3**	0.588^a^	0.432^a.b^	0.568^a.b^	0.493^a.b^	0.398^b^	0.024	0.042	0.063	0.274
**22:0**	0.152^a.b^	0.115^b^	0.172^a^	0.159^a.b^	0.134^a.b^	0.026	0.037	0.686	0.868
**22:4n-6**	1.617	1.443	1.783	1.539	1.824	0.062	0.236	0.853	0.648
**22:5n-3**	0.810	0.766	0.919	0.878	0.965	0.083	0.100	0.676	0.670
**22:5n-6**	2.390	2.104	2.405	2.026	2.657	0.038	0.485	0.475	0.378
**22:6n-3**	2.051	2.045	2.184	2.027	2.600	0.090	0.210	0.491	0.354
**∑ SFA**	36.011	37.586	37.546	37.194	36.325	0.285	0.290	0.120	0.195
**∑ MUFA**	25.862	25.208	22.830	25.843	23.348	0.434	0.059	0.170	0.577
**∑ PUFA**	30.374	28.742	32.057	29.112	33.227	0.643	0.116	0.809	0.624
**∑ OBCFA**	3.466	3.444	3.316	3.568	3.169	0.076	0.525	0.650	0.949
**∑ PUFA n-6**	26.061	24.906	27.762	25.192	28.780	0.563	0.118	0.687	0.635
**∑ PUFA n-3**	3.417	3.007	3.555	3.056	3.619	0.098	0.143	0.676	0.815
**PUFA n-6/n-3**	7.672	8.765	7.902	8.338	7.990	0.152	0.180	0.145	0.634

SFA: saturated fatty acids; MUFA: monounsaturated fatty acids; PUFA: polyunsaturated fatty acids; OBCFA: odd and branched chain fatty acids; SEM: Standard error of mean.

^1^Dietary treatments: CO: Control; CH: Chestnut extract; TA: Tara extract; GA: Gambier extract; MI: Mimosa extract.

^2^Contrasts: Tannins (overall effect of tannin: CON vs. CH + TA + GA + MI); Tannin type (effect of HT vs. CT: CH + TA vs. GA + MI).

Dietary treatment means within a row with different superscripts differ at a significance of P ≤ 0.05.

Table 5 reports data on the FA composition of intramuscular fat (IMF). Dietary treatment, tannin supplementation and tannin type did not affect total SFA, MUFA, PUFA, the sum of PUFA n-3 and PUFA n-6 and the PUFA n-6 to n-3 ratio. Only the sum of OBCFA increased in tannin-fed lambs in comparison to CO (*P* = 0.007). Within the class of OBFCA, 15:0 and 17:0*anteiso* were lower in the meat of tannin-fed lambs in comparison with the CO (*P* ≤ 0.05). Regarding tannin type, dietary HT increased (*P* ≤ 0.05) 14:0i*so*, 15:0*iso*; 17:0*iso* and tended to reduce 17:0 (*P* = 0.069) in comparison with CT. Moreover, regarding the effect of dietary treatment on individual OBCFA, 17:0 was higher in the IMF of the CO lambs as compared to CH (*P* = 0.023); moreover 14:0*iso* (*P* = 0.002), 17:0*iso* (*P* = 0.020) were higher in the CH lambs as compared to GA; 14:0*iso* was higher in the CH in comparison with MI (*P* = 0.029). As for 18-carbon fatty acids involved in the BH, no effect of dietary treatment, tannin supplementation or type (*P* > 0.05) was found for 18:0, 18:2*c*9*c*12 and 18:3c*9c*12*c*15. Conversely, dietary tannins reduced 18:2*c*9*t*11 in comparison to CO group (*P* = 0.023). Tannin supplementation and dietary treatments affected 18:1*t*11. In particular, tannin supplementation reduced 18:1*t*11 (*P* = 0.025) in comparison to CO, with CT tending to reduce 18:1*t*11 more than HT (*P* = 0.078). Regarding differences between groups, a lower proportion of 18:1*t*11 was found in the IMF of GA lambs in comparison with CO (*P* = 0.003) and CH (*P* = 0.039). Within the identified trans 18:1 isomers, 18:1*t*6+*t*7+*t*8 and 18:1*t*10 were lower in the HT than CT fed lambs (*P* = 0.037 and *P* = 0.010, respectively), moreover, they were lower in CH group as compared to CO (*P* = 0.004 and *P* = 0.005, respectively) and MI (*P* < 0.001 and *P* < 0.001, respectively). Finally, 18:1*t*6+*t*7+*t*8 and 18:1*t*10 were found at lower proportion in the GA group in comparison with MI (*P* = 0.019 and *P* = 0.023, respectively). The 18:1*t*9 was lower in the GA meat as compared to CO and MI (*P* = 0.040 and *P* < 0.001, respectively), and in the CH group in comparison with MI (*P* = 0.003). Regarding other PUFA in meat, 18:2*t*8*c*13 and 18:2*t*9*c*12 were affected by tannin supplementation and differed between the five dietary treatments. In particular, 18:2*t*8*c*13 was lower in the meat of CO than tannin-fed groups (P = 0.002) which, conversely, had lower proportions of 18:2*t*9*c*12 (P = 0.007). The meat of HT-fed groups showed a greater proportion of 18:2*t*9*c*12 than CT (P = 0.016). Moreover, 18:2*t*8*c*13 significantly differed between CO and CH (P < 0.001) while, 18:2*t*9*c*12 was higher in CO group in comparison with GA and MI (*P* = 0.001 and *P* = 0.002, respectively) as well as in the CH group as compared to GA and MI (*P* < 0.001 and *P* < 0.001, respectively).

**Table 5:** Effect of different tannin extracts on meat fatty acid composition (g/100g total fatty acids).

	Diet^1^					SEM	*p* value	Contrast^2^	
	CO	CH	TA	GA	MI			Tannins	Tannin type
**IMF (g/100 meat)**	2.075	1.853	1.862	1.770	1.984	0.068	0.672	0.240	0.749
**10:0**	0.120	0.124	0.123	0.114	0.136	0.004	0.580	0.710	0.878
**12:0**	0.128	0.144	0.121	0.110	0.130	0.005	0.185	0.874	0.232
**14:0**	2.896	3.013	2.832	2.697	2.755	0.066	0.600	0.680	0.193
**14:0*iso***	0.018^a.b^	0.022^a^	0.018^a.b^	0.014^b^	0.016^b^	0.001	0.010	0.683	0.004
**14:1*c*9**	0.111	0.118	0.109	0.103	0.101	0.004	0.627	0.737	0.163
**15:0**	0.357	0.294	0.312	0.304	0.309	0.009	0.181	0.015	0.847
**15:0*iso***	0.068	0.070	0.061	0.054	0.057	0.002	0.058	0.148	0.040
**15:0*anteiso***	0.108	0.107	0.107	0.095	0.096	0.003	0.442	0.377	0.109
**16:0**	23.60	23.29	23.58	23.05	23.10	0.196	0.856	0.501	0.436
**16:0*iso***	0.121	0.127	0.116	0.104	0.114	0.003	0.102	0.470	0.056
**16:1*c*7**	0.310	0.309	0.270	0.275	0.268	0.006	0.044	0.063	0.204
**16:1*c*9**	1.772	1.666	1.689	1.694	1.658	0.031	0.831	0.247	0.978
**17:0**	1.152^a^	0.877^b^	0.972^a.b^	1.017^a.b^	1.057^a.b^	0.029	0.042	0.022	0.069
**17:0*iso***	0.366^a.b^	0.391^a^	0.349^a.b^	0.328^b^	0.352^a.b^	0.007	0.050	0.538	0.060
**17:0*anteiso***	0.486	0.431	0.431	0.442	0.449	0.008	0.222	0.024	0.430
**17:1*c*9**	0.708	0.510	0.586	0.637	0.621	0.022	0.058	0.031	0.073
**18:0**	12.13	12.96	12.40	12.80	13.01	0.159	0.364	0.109	0.560
**18:1*c*9**	38.78	37.68	37.19	38.47	38.48	0.380	0.681	0.408	0.248
**18:1*c*11**	1.575	1.428	1.546	1.655	1.407	0.040	0.240	0.531	0.635
**18:1*c*12**	0.435^a^	0.416^a.b^	0.332^c^	0.344^b.c^	0.329^c^	0.012	0.003	0.008	0.108
**18:1*c*13**	0.108	0.098	0.097	0.121	0.121	0.004	0.144	0.875	0.006
**18:1*c*14**	0.049	0.044	0.040	0.043	0.042	0.001	0.301	0.040	0.926
**18:1*t*5**	0.015	0.014	0.016	0.013	0.016	0.001	0.556	0.817	0.691
**18:1*t*6+*t*7+*t*8**	0.283^a.b^	0.212^c^	0.256^a.b.c^	0.243^c^	0.302^a^	0.008	0.002	0.164	0.037
**18:1*t*9**	0.264^a.b^	0.222^b.c^	0.242^b.c^	0.209^c^	0.296^a^	0.007	<0.001	0.280	0.230
**18:1*t*10**	1.579^a.b^	0.791^c^	1.199^b.c^	1.121^b.c^	1.759^a^	0.083	<0.001	0.092	0.010
**18:1*t*11**	0.753^a^	0.669^a^	0.608^a.b^	0.458^b^	0.612^a.b^	0.029	0.020	0.025	0.078
**18:2*c*9*c*12**	6.846	8.110	8.354	7.667	7.354	0.288	0.518	0.173	0.278
**18:2*c*9*t*11**	0.434	0.379	0.365	0.304	0.355	0.014	0.074	0.023	0.169
**18:2*t*10*c*12**	0.024	0.033	0.033	0.025	0.029	0.002	0.417	0.216	0.192
**18:2*t*8*c*13**	0.065^c^	0.196^a^	0.100^b.c^	0.155^a.b^	0.140^a.b^	0.011	<0.001	0.002	0.977
**18:2*t*9*c*12**	0.065^a^	0.066^a^	0.044^b^	0.043^b^	0.044^b^	0.002	<0.001	0.007	0.016
**18:2*t*9*c*13**	0.267	0.362	0.305	0.293	0.294	0.011	0.070	0.100	0.063
**18:3*c*6*c*9*c*12**	0.055	0.063	0.062	0.066	0.058	0.003	0.824	0.364	0.872
**18:3*c*9*c*12*c*15**	0.525	0.570	0.550	0.534	0.542	0.009	0.589	0.299	0.313
**20:0**	0.093	0.100	0.098	0.097	0.097	0.002	0.769	0.242	0.554
**20:3n-6**	0.153	0.190	0.205	0.186	0.142	0.011	0.359	0.343	0.200
**20:3n-3**	0.020	0.023	0.021	0.019	0.022	0.001	0.706	0.669	0.515
**20:4n-6**	1.404	1.800	2.102	1.988	1.476	0.145	0.484	0.247	0.524
**20:5n-3**	0.082	0.091	0.135	0.097	0.064	0.011	0.294	0.605	0.199
**22:0**	0.037	0.031	0.039	0.044	0.031	0.002	0.369	0.953	0.581
**22:4n-6**	0.131	0.177	0.206	0.202	0.163	0.015	0.524	0.146	0.803
**22:5n-3**	0.221	0.286	0.348	0.334	0.230	0.026	0.434	0.254	0.576
**22:5n-6**	0.036	0.046	0.056	0.059	0.038	0.005	0.556	0.307	0.851
**22:6n-3**	0.050	0.079	0.106	0.097	0.054	0.009	0.176	0.141	0.420
**∑ SFA**	39.00	39.66	39.20	38.90	39.25	0.307	0.954	0.757	0.640
**∑ MUFA**	46.74	44.18	44.18	45.39	46.01	0.416	0.219	0.095	0.107
**∑ PUFA**	10.38	12.47	12.99	12.07	11.00	0.488	0.453	0.167	0.291
**∑ OBCFA**	2.676	2.318	2.365	2.357	2.450	0.045	0.091	0.007	0.526
**∑ PUFA n-6**	8.623	10.386	10.980	10.168	9.231	0.448	0.503	0.180	0.344
**∑ PUFA n-3**	0.898	1.048	1.161	1.082	0.911	0.051	0.444	0.257	0.380
**PUFA n-6/n-3**	9.534	9.920	9.834	9.493	10.134	0.234	0.910	0.613	0.912
**Indexes**									
** DI 14:0**	0.037	0.036	0.037	0.036	0.037	0.001	0.995	0.945	0.616
** DI 16:0**	0.070	0.066	0.067	0.067	0.069	0.001	0.930	0.442	0.648
** DI of 17:0** ^**3**^	0.379	0.366	0.374	0.377	0.375	0.003	0.572	0.539	0.476
** AI** ^**4**^	0.628	0.630	0.625	0.618	0.607	0.010	0.808	0.750	0.296
** TI** ^**5**^	1.268	1.273	1.257	1.278	1.263	0.019	0.947	0.990	0.840

SFA: saturated fatty acids; MUFA: monounsaturated fatty acids; PUFA: polyunsaturated fatty acids; OBCFA: odd and branched chain fatty acids; SEM: Standard error of mean.

^1^Dietary treatments: CO: Control; CH: Chestnut extract; TA: Tara extract; GA: Gambier extract; MI: Mimosa extract.

^2^Contrasts: Tannins (overall effect of tannin: CON vs. CH + TA + GA + MI); Tannin type (effect of HT vs. CT: CH + TA vs. GA + MI).

Dietary treatment means within a row with different superscripts differ at a significance of P ≤ 0.05.

^3^Desaturation index of 17:0 calculated as 17:1 *c*9/(17:0+17:1*c*9).

^4^Atherogenic index calculated as: (C12:0 + 4×C14:0 + C16:0) / (MUFA + PUFAn-6 + PUFAn-3) (Ulbricht and Southgate, 1991).

^5^Thrombogenic index calculated as: (C14:0 + C16:0 + C18:0)/(0.5×MUFA + 0.5×PUFAn-6 + 3×PUFAn-3 + PUFAn-3/PUFAn-6) (Ulbricht and Southgate,1991).

### Multivariate approach to fatty acid profile

**Fig 1:**
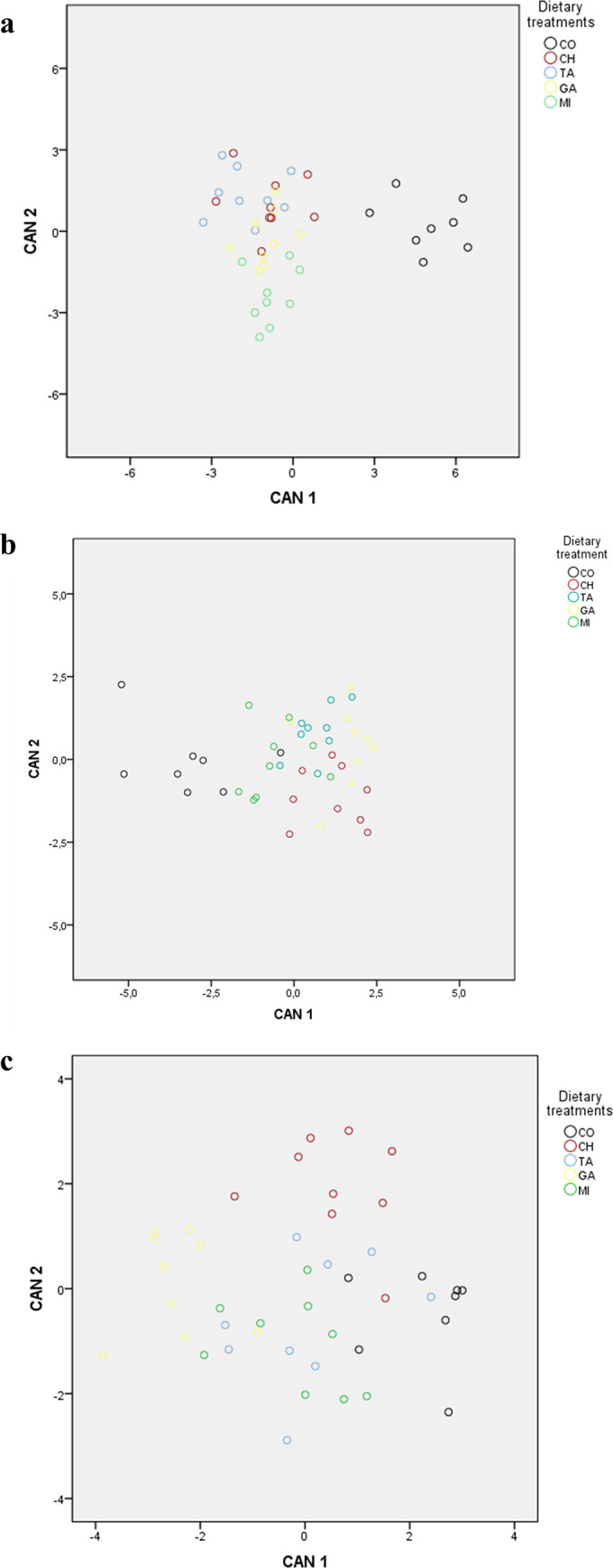
Discrimination of the dietary treatments achieved by plotting the first two canonical functions obtained from (a) the dataset of rumen content fatty acid, (b) the dataset of liver fatty acids and (c) the dataset of IMF fatty acids after the stepwise procedure. Dietary treatments: CO: Control; CH: Chestnut extract; TA: Tara extract; GA: Gambier extract; MI: Mimosa extract.

From the entire dataset of the rumen content, eight FAs were retained at the end of the stepwise selection (Table 6) and were linearly combined to obtain four canonical discriminant functions (CAN), which together explained the total variance of the dataset. The multivariate structure could be appropriately described by the first two CAN, explaining 70.0% and 22.7% of the variance, respectively. Therefore, the scatterplot (Fig 1A) represents the distribution of the rumen content samples sample in the multivariate space, with CAN 1 as the X axis and the CAN 2 as the Y axis. The CAN 1 alone allowed discrimination between the CO and tannins groups. The CAN 2 tended to separate the CH lambs from all the other groups with some overlaps with the MI or GA groups. After cross-validation, 66% of cases were correctly assigned. Regarding wrongly assigned cases: only one CO animal was misclassified and assigned to CH; one and four CH cases were assigned to TA and GA, respectively; three TA cases were assigned to the other HT group (CH); two cases of MI were assigned to the other CT group (GA). Table 6 reports the standardized coefficients of the variables describing the total canonical structure for the rumen content. The magnitude (absolute value) of the standardized coefficients assigned to each variable is a measure of the relative contribution of the variable itself to the discriminating capacity of the whole multivariate function: the greater the value of the coefficient, the higher the contribution of the variable to the discrimination. The CAN 1 was mainly influenced by 17:0*anteiso*, 18:1*t*10, 18:2*t*8*c*13; 18:2*c*9*c*12 and 22:0; while 17:0*anteiso*, 18:1*t*5, 18:2*t*9*c*12, 18:2*t*10*c*12 and 18:2*c*9*c*12 accounted most in the CAN 2.

**Table 6:** Standardized coefficients of each canonical function and explained variance.

	CAN 1	CAN 2	CAN 3	CAN 4
**Rumen content**				
** 17:0*anteiso***	0.774	0.695	-0.109	-0.186
** 18:1 t5**	-0.210	-0.654	0.300	-0.103
** 18:1 t10**	1.138	-0.342	0.387	-0.200
** 18:2 t8c13**	-0.657	-0.090	-0.491	0.460
** 18:2 t9c12**	-0.196	0.693	0.790	0.320
** 18:2 t10c12**	0.113	-1.116	0.271	0.124
** 18:2 c9c12**	-0.678	0.781	-0.460	-0.665
** 22:0**	1.205	0.242	-0.006	0.594
** Explained variance (%)**	70.0	22.7	6.9	0.4
**Liver**				
** 18:1 t10**	-0.741	0.176	0.548	0.386
** 18:2 t9c13**	0.885	0.207	1.391	0.401
** 18:2 c9t11**	-1.287	-0.069	-0.487	-0.362
** 18:3 c9c12c15**	1.082	0.683	-0.065	0.762
** 22:0**	0.286	0.966	0.621	0.762
** Explained Variance (%)**	79.1	12.5	7.4	1.4
**Meat**				
** 16:0*iso***	0.502	0.393	0.515	-0.425
** 18:1 c14**	-1.028	-0.274	-1.216	1.042
** 18:1 t9**	0.398	-0.572	0.558	0.504
** 18:2 c9t11**	1.303	-0.102	0.451	-0.682
** 18:2 t8c13**	-0.627	0.601	0.565	0.293
** 18:2 t9c12**	0.822	0.565	-0.167	0.260
** Explained variance (%)**	55.6	27.2	13.2	4.1

The same procedure described for the rumen fatty acid was used separately for liver and meat fatty acids datasets. For liver, 5 fatty acids were retained from the dataset after stepwise selection (Table 6) and combined to obtain four CAN. The first two CAN explained 79.1.6% and 12.5% of the variance, respectively. Fig 1B represents the distribution of the livers sample in the multivariate space. After cross-validation, 59.1 of cases were correctly assigned to the group of origin. However, only two cases of CO liver were misclassified and assigned to MI. Table 6 reports the standardized coefficients of the variables describing the total canonical structure for the liver. The CAN 1 was mainly influenced by 18:2*c*9*t*11, 18:2*t*9*c*13 and 18:3*c*12*c*15*c*18; while 22:0 and 18:3*c*12*c*15*c*18 accounted most in the CAN 2.

From the dataset of the meat fatty acids, six were retained at the end of the stepwise selection (Table 6). The first two CAN explained 55.6% and 27.1% of the variance, respectively. Fig 1C represents the distribution of the lambs in the multivariate space. Only 56.8% of cases were correctly assigned after cross-validation. Table 6 reports the standardized coefficients of the variables describing the total canonical structure for the meat. The CAN 1 was mainly influenced by 18:1*c*14, 18:2*c*9*t*11 and 18:2*t*9*c*12; while 18:1*t*9 18:2*t*8*c*13 and 18:2*t*9*c*12 accounted most in the CAN 2.

## Discussion

### Animal performance

Literature reports that the inclusion of tannins in the diet for ruminants may depress voluntary feed intake, thus impairing animal performance [30, 31]. Several possible mechanisms have been considered, alone or in association, to explain how tannins influence DMI. Among these, the reduction of the feed palatability would be due to the astringency perception induced by a direct reaction of tannins with the taste receptors or through the interaction with the tannin-binding salivary proteins [7]. The reduction of DMI has been usually observed at tannin concentrations that exceed 50 g per kg of diet, while lower doses would not be unfavourable and may enhance animal health and production [14, 32]. In small ruminants, the negative effects of tannins would be mitigated by adaptation mechanisms, such as the production of salivary proteins able to neutralize the astringency of tannins [33]. Also, HT and CT may result in different effects because: *i*) HT would be more susceptible to be hydrolysed than CT in the rumen and release monomers with toxic effects [3]; *ii*) the complexes that CT create with proteins and other nutrients are stable in a wider pH range than HT [34, 35]. Based on this knowledge and that of previous findings revealing specific antimicrobial activity of different tannin extracts [19], we have evaluated the response of animal performance and fatty acids in the rumen content and meat of growing lambs fed commercial sources of HT (chestnut and tara) and CT (mimosa and gambier). Also, within HT and CT classes, we have tested whether different sources of plant extracts, each characterised by the occurrence of chemically different tannins, may elicit different responses.

Our findings partially contrast with the literature. Indeed, though the level of tannin inclusion in the diet was lower than the threshold of 50 g per kg of diet, it reduced the DMI in comparison with the CO group. The lower DMI observed in lambs fed HT compared to those fed CT, was mainly due to the effect of the CH group, which showed the least DMI and growth performance among the treatments. Conversely, none of the investigated productive parameters differed between CO and TA, GA and MI. These findings are particularly interesting because they suggest several considerations that challenge some accepted generalizations on the use of tannins in ruminant diets. First, the lack of different performance between TA lambs (supplemented with a source of HT) and CT-fed lambs may indicate that general rules regarding the effects of hydrolysable or condensed tannins on animal performance should be avoided or at least carefully considered. Second, the response to the administration of tannin extracts belonging to the same class of compounds (in our case HT) may differ according to the chemical characteristics of tannins or tannin source. It has been reported that different tannins may have a varying degree of affinity with salivary proteins in sheep [36]. Moreover, beside the botanical difference of the plants, the CH and TA extracts enriched the diet with ellagitannins and gallotannins, respectively [37]. Deschamps et al. [38, 39] report that hydrolysable tannins contained in CH and TA extracts are susceptible to biodegradation by bacterial tannase in the rumen with the production of several intermediates along with the enzymatic reactions, to form acetate and butyrate as end products. Though ruminal biodegradation intermediates of tannins were not investigated in the present paper, it could be stressed that the difference observed between CH and TA groups could be in part linked to the different toxicity of intermediate products. The last consideration is related to the ability of tannins to reduce nutrient availability. In our study, CH showed ADG lower than all the other groups. Noteworthy, this result seems to be due to the lower DMI rather than other factors, such as the reduction of protein digestibility. As observed in the companion paper, the level of ammonia in the rumen was not affected by dietary treatment, tannin supplementation nor tannin type [19]. Rumen ammonia is related with the proteolytic bacterial metabolism, which may represent a limiting factor for the rate of growth in young animals.

### Fatty acids of rumen, liver and meat

Similar to animal performance, our results on the effect of dietary tannins on the fatty acid composition of rumen content seem to point a different direction in comparison with that indicated by the literature. Most of the previous *in vitro* and *in vivo* studies agree that tannins may impair the progressive saturation of PUFA to 18:0 and, as a consequence, increase the outflow of health-promoting fatty acids that can be absorbed, further metabolized and accumulated in the tissues [40, 41]. In the rumen, an increase of PUFA would be observed when the first steps on the onset of the BH are inhibited [42–44]. On the contrary, the inhibition of the BH at different steps along the pathways might result in a lower formation of 18:0 and greater accumulation of intermediates such as 18:2*c*9*t*11 and 18:1*t*11 as well as other *trans* 18:1 isomers [45–47]. It should be mentioned that the characteristics of the basal diet seem to play an important role in determining the effects of tannins on rumen BH. The 18:1*t*11 accounts up to 70% of total *trans* 18:1 isomers in forage-fed ruminants [48], while the progressive replacement of forages with concentrates increases 18:1*t*10 at the expense of 18:1*t*11 due to a changed BH pathway [49]. Vasta et al. [14] observed that condensed tannins from quebracho hampered this phenomenon when they were administered to concentrate-fed lambs compared to herbage-fed animals. Moreover, the capability of the different ruminant species to tolerate plant polyphenols represents an additional factor that deserve consideration when the effect of tannins on BH is investigated. Frutos et al. [8] reviewed differences between cows and ewes in the accumulation of 18:1 isomers *in vitro*.

Apparently, in our study, the administration of dietary tannins was ineffective to interfere with the different steps of the biohydrogenation, as no significant difference was observed in the rumen for total PUFA, 18:2*c*9*c*12, 18:3*c*9*c*12*c*15, 18:2*c*9*t*11, 18:1*t*11 and 18:0. However, we found that the proportion of 18:1*t*10 was lower in the rumen of tannin-fed lambs. This reduction was not in favour of a greater accumulation of 18:1*t*11 in the rumen, suggesting that tannin supplementation could have specifically reduced alternative pathways of BH, with HT tending to be more effective than CT. These results are not easily explainable in relation to what we have found in the companion paper. In that study [19], HT-fed lambs showed a lower ruminal pH than CT-fed lambs, which is a condition that favours the synthesis of 18:1*t*10 in the rumen [49]. Also, it has been reported that different bacterial species may be responsible for different BH pathways. In the companion paper, Salami et al. [19] observed that the supplementation of tannins did not substantially alter the bacterial community and relative abundance in the rumen of the same lambs used in the present. Only *Fibrobacteres*, representing a minority group of total bacteria in the rumen, were significantly lower in the tannin-receiving groups in comparison with the CO. However, it should be noticed that the relative abundance does not necessarily determine the functional activity of a bacteria group in the rumen. In other studies, changes in *Fibrobacter* have been linked to the modification in BH [50]. Also, Salami et al. [19] reported treatment differences in bacterial genera such as Prevotella and Ruminobacter and these genera have been suggested to play significant roles in BH. The lack of a straightforward effect on rumen microbial community seems in agreement with the proportion of OBCFA observed in the rumen. The presence of OBCFA exclusively arises from microbial metabolism and a variation in OBCFA is expected to reflect changes in the relative abundance of the specific bacterial population [51]. In particular, Vlaemink et al. [52] reported that fibre-digesting bacteria are richer in even and/or odd- *iso*-fatty acids than amylolytic bacteria, which are characterized by the predominance of 15:0 and 15:0*anteiso*. In our study, total and individual OBCFA did not follow a specific trend that could help to unveil possible effects of tannins and tannin type on the microbial community useful to explain the results observed for 18:1*t*10.

Liver could be considered as an intermediate step along the metabolic pathway of fatty acids that leads to their accumulation in the muscle, as they may undergo partial transformation in this organ before being further metabolized in the tissues. The fatty acid composition of meat and liver partially agrees with the results observed in the rumen content. In particular, the lack of effect of the investigated factors on the main substrates of the BH (18:2*c*9*c*12, 18:3*c*9*c*12*c*15), total PUFA and end-product of the process (18:0) would confirm that, in our experimental conditions, the addition of tannins or the type of tannins supplied to growing lambs did not protect dietary PUFA from BH and did not favour the accumulation of these healthful fatty acids. In accordance with the rumen content, 18:1*t*10 was greater in the liver and in the meat (even if only as a statistical tendency) from CO group than tannin-receiving groups, moreover, it was greater in the liver and meat from CT-fed lambs than HT-fed lambs. A similar effect of tannin supplementation was observed in the liver for 18:*1t9*, 18:*1t*11 and 18:1*c*9*t*11 greater in CO than in tannin-supplemented groups. These data may suggest that an impairment of the intermediate steps of the biohydrogenation process due to the inclusion of tannins in the diet of lambs may have occurred, and in particular the inhibition of the alternative pathways of the BH, and that HT could have been more effective than CT. Moreover, some inconsistencies between fatty acids in the rumen content, liver and meat seem to indicate a wider effect of the tannins. Discrepancies between the fatty acid profile of rumen content, liver and meat could depend on several factors, such as the collection technique or the sampling time. In this study the rumen fatty acids describe the specific moment of slaughter, while liver and meat fatty acids result from absorption mechanisms and further lipid metabolism in liver and tissues. Therefore, liver and meat fatty acid composition may add useful information on fatty acid metabolism. Differing from rumen content, 18:1*t*11 followed the same trend as the 18:1*t*10. Due to the reasons explained above, usually, these two fatty acids are negatively correlated. In the present study, the contemporary reduction of both 18:2*c*9*t*11 and 18:1*t*10 could indicate that the administration of dietary tannins may have caused a general reduction of BH extent, though with a magnitude lower than reported in the literature. It should be stressed that the characteristics of the basal diet may play an important role in favouring the effects of dietary tannins. For example, Jeronimo et al [12] and Frutos et al. [8] highlighted that the supplementation of PUFA-rich oils may enhance the effect of tannins on ruminal BH. In the present trial, the diet given to the animal was not added with PUFA, therefore, it cannot be excluded that a greater availability of PUFA in the rumen could have hampered the effect of tannins on BH.

### Multivariate approach to fatty acid profile

Individual fatty acids significantly differed between treatments both in the rumen content and meat. However, the univariate approach did not allow identifying specific patterns of fatty acid metabolism due to the tannin extracts. Differing from the univariate, the multivariate statistics can be implemented to assess the combined role of the individual variables of a dataset in highlighting overall patterns within the dataset. For example, among the multivariate approaches, the canonical discriminant analysis may allow to discriminate between subjects (samples) belonging to different groups. The canonical discriminant analysis can be coupled with a stepwise procedure that identifies the variables of the dataset having the biggest discriminating ability. This approach has been previously applied to different datasets to discriminate between meat or cheese samples differing for the geographical origin, breed or production system [29, 53, 54]. In the present study, the same approach was used: *i*) to assess whether the multivariate structure of the FA profile was able to reveal overall patterns able to discriminate the dietary treatments, *ii*) to identify the fatty acids characterized by a greater discriminating power both in the rumen content and meat. After the stepwise procedure, 8, 5 and 6 fatty acids were retained from the respective datasets of rumen content, liver fat and IMF and the retained fatty acids were linearly combined into 4 canonical discriminant functions for each biological matrix. All the fatty acids with a high standardized coefficient included in the two first CAN for each of the three datasets arise from rumen microbial metabolism or BH, except for 22:0. However, most of them were not significantly affected by the dietary treatments and only 66% (rumen content), 59% (liver) and 57% (meat) of cases were correctly assigned to the original experimental group after cross-validation. These results seem to confirm that the dietary administration of the four tannin extracts did not produced specific patterns describing their effects on fatty acid metabolism. A similar finding was reported by Morales et al. [55] after a regression analysis conducted on 12 different experiments to investigate the response of meat fatty acids to tannins supplementation. The authors reported that, tannins were associated only with the reduction of 18:0. Nevertheless, it should be highlighted for all the three main districts implicated in the fatty acid metabolism (i.e., rumen content, liver and meat), the CO group was well discriminated from the tannin-fed groups, with maximum 2 cases from the CO group being misassigned to a tannin-supplemented group (Fig 1). Together with the results of the univariate ANOVA, the multivariate analysis showed that dietary tannins exerted a general modification along the whole BH process, though it was not possible to identify specific patterns for each of the administered extracts.

## Conclusions

Our study confirmed that the dietary administration of tannins may have an effect in determining the fatty acid profile of the rumen content and meat in lambs. However, it was not possible to clearly describe specific mechanisms of action of the individual supplemented tannin extracts on precise steps of the fatty acid metabolism, as previous studies supposed. Nevertheless, in our study, the multivariate structure of the fatty acid profile of rumen content and meat allowed the discrimination of the CO treatment from the other groups. Interestingly, the fatty acids showing the greater discriminant power arise from microbial metabolism and the principal or alternative BH pathways. These results may give new perspectives in investigating the effect of tannins on BH. From one side, our study further confirms the impact that dietary tannins may exert on the rumen BH. From the other side, it provides clues that tannins can have a generalized influence on BH rather than on specific steps.

## References

[1] Patra AK, Saxena J. Dietary phytochemicals as rumen modifiers: a review of the effects on microbial populations. Antonie Van Leeuwenhoek. 2009;96: 363–375. doi: 10.1007/s10482-009-9364-1 19582589

[2] Reed JD. Nutritional toxicology of tannins and related polyphenols in forage legumes. J Anim Sci. 1995;75: 1516–1528. doi: 10.2527/1995.7351516x 7665384

[3] Makkar HPS. Effects and fate of tannins in ruminant animals, adaptation to tannins, and strategies to overcome detrimental effects of feeding tannin-rich feeds. Small Rum Res. 2003;49: 241–256.

[4] McCann JC, Wickersham TA, Loor JJ. High-throughput methods redefine the rumen microbiome and its relationship with nutrition and metabolism. Bioinform Biol Insights. 2014;8: 109–125. doi: 10.4137/BBI.S15389 24940050 PMC4055558

[5] Vasta V, Daghio M, Cappucci A, Buccioni A, Serra A, Viti C, et al. Invited review: Plant polyphenols and rumen microbiota responsible for fatty acid biohydrogenation, fiber digestion, and methane emission: Experimental evidence and methodological approaches. J Dairy Sci. 2019;102: 3781–3804. doi: 10.3168/jds.2018-14985 30904293

[6] Mcleod MN. Plant tannins—Their role in forage quality. Nutr Abstr Rev. 1974;44: 803–812.

[7] Mueller-Harvey I. Unravelling the conundrum of tannins in animal nutrition and health. J Sci Food Agric. 2006;86: 2010–2037.

[8] Frutos P, Hervás G, Natalello A, Luciano G, Fondevila M, Priolo A, et al. Ability of tannins to modulate ruminal lipid metabolism and milk and meat fatty acid profile. Anim. Feed Sci. Technol. 2020;269: 114623.

[9] Vasta V, Makkar HPS, Mele M, Priolo A. Ruminal biohydrogenation as affected by tannins in vitro. Br J Nutr. 2009;102: 82–92. doi: 10.1017/S0007114508137898 19063768

[10] Carreño D, Hervás G, Toral PG, Belenguer A, Frutos P. Ability of different types and doses of tannin extracts to modulate in vitro ruminal biohydrogenation in sheep. Anim Feed Sci Technol. 2015;202: 45–51.

[11] Natalello A, Luciano G, Morbidini L, Valenti B, Pauselli M, Frutos P, et al. Effect of feeding pomegranate byproduct on fatty acid composition of ruminal digesta, liver, and muscle in lambs. J Agr Food Chem. 2019;67: 4472–4482. 30929432 10.1021/acs.jafc.9b00307

[12] Jeronimo E, Alves SP, Dentinho MT, Martins SV, Prates JA, Vasta V, et al. Effect of grape seed extract, Cistus ladanifer L., and vegetable oil supplementation on fatty acid composition of abomasal digesta and intramuscular fat of lambs. J Sci Food Agric. 2010;58: 10710–10721. 10.1021/jf1021626 20831248

[13] Waghorn GC, McNabb WC. Consequences of plant phenolic compounds for productivity and health of ruminants. Proc Nutr Soc. 2003;62: 383–392. doi: 10.1079/pns2003245 14506885

[14] Vasta V, Mele M, Serra A, Scerra M, Luciano G, Lanza M, et al. Metabolic fate of fatty acids involved in ruminal biohydrogenation in sheep fed concentrate or herbage with or without tannins. J Anim Sci. 2009;87: 2674–2684. doi: 10.2527/jas.2008-1761 19395521

[15] Jerónimo E, Alfaia CM, Alves SP, Dentinho MT, Prates JA, Vasta V, et al. Effect of dietary grape seed extra, Cistus ladanifer L. in combination with vegetable oil supplementation on lamb meat quality. Meat Sci. 2012;92: 841–847. doi: 10.1016/j.meatsci.2012.07.011 22885021

[16] Toral P, Hervàs G, Bichi E, Belenguer A, Frutos P. Effect of the inclusion of quebracho tannins in diet rich in linoleic acid on milk fatty acid composition in dairy ewes. J Dairy Sci. 2013;96: 431–439. doi: 10.3168/jds.2012-5622 23164228

[17] Costa M, Alves SP, Cappucci A, Cook SR, Duarte A, Caldeira RM, et al. Effects of condensed and hydrolyzable tannins on rumen metabolism with emphasis on the biohydrogenation of unsaturated fatty acids. J Agr Food Chem. 2018;66: 3367–3377. doi: 10.1021/acs.jafc.7b04770 29494146

[18] Buccioni A, Pauselli M, Viti C, Minieri SA, Pallara G, Roscini V, et al. Milk fatty acid composition, rumen microbial population and animal performances in response to diets rich in linoleic acid supplemented with chestnut or quebracho tannins in dairy ewes. J Dairy Sci. 2015;98: 1145–1156. doi: 10.3168/jds.2014-8651 25434333

[19] Salami SA, Valenti B, Bella M, O’Grady MN, Luciano G, Kerry JP, et al. Characterisation of the ruminal fermentation and microbiome in lambs supplemented with hydrolysable and condensed tannins. FEMS Microbiol Ecol 2018;94: fiy061. doi: 10.1093/femsec/fiy061 29648587

[20] Van Soest PJ, Robertson JB, Lewis BA. Methods for dietary fibre, neutral detergent fiber, and nonstarch polysaccharides in relation to animal nutrition. J Dairy Sci. 1991;74: 3583–3597. doi: 10.3168/jds.S0022-0302(91)78551-2 1660498

[21] AOAC (Association of Official Analytical Chemists) Official methods of analysis (16th edition). Washington: AOAC; 1995.

[22] Valenti B, Luciano G, Morbidini L, Rossetti U, Codini M, Avondo M, et al. Dietary pomegranate pulp: effect on ewe milk quality during late lactation. Animals. 2019;9: 283. doi: 10.3390/ani9050283 31137876 PMC6562843

[23] Alves SP, Francisco A, Costa M, Santos-Silva J, Bessa RJ. Biohydrogenation patterns in digestive contents and plasma of lambs fed increasing levels of a tanniferous bush (*Cistus ladanifer* L.) and vegetable oils. Anim Feed Sci Technol. 2017;225: 157–172.

[24] Folch J, Lees M, Stanley GHSA. simple method for the isolation and purification of lipids from animal tissue. J Biol Chem. 1957;226: 497–509. 13428781

[25] Christie WW. A simple procedure for rapid transmethylation of glycerolipids and cholesteryl esters. J Lipid Res. 1982;23: 1072–1075. 6897259

[26] Valenti B, Luciano G, Pauselli M, Mattioli S, Biondi L, Priolo A, et al. Dried tomato pomace supplementation to reduce lamb concentrate intake: Effects on growth performance and meat quality. Meat Sci. 2018;145: 63–70. doi: 10.1016/j.meatsci.2018.06.009 29906738

[27] Kramer JK, Hernandez M, Hernandez CC, Kraft J, Dugan ME. Combining results of two GC separations partly achieves determination of all cis and 16:1, 18:1, 18:2 and 18:3 except CLA isomers of milk fat as demonstrated using Ag-ion SPE fractionation. Lipids. 2008;43: 259–273. doi: 10.1007/s11745-007-3143-4 18214567

[28] Alves SP, Bessa RJB. Identification of cis-12,cis-15 octadecadienoic acid and other minor polyenoic fatty acids in ruminant fat. Eur J Lipid Sci Technol. 2007;109: 879–883.

[29] Valenti B, Biondi L, Campidonico L, Bontempo L, Luciano G, Di Paola F, et al. Changes in stable isotope ratios in PDO cheese related to the area of production and green forage availability. The case study of Pecorino Siciliano. Rapid Commun Mass Spectrom. 2017;31: 737–744. doi: 10.1002/rcm.7840 28220554

[30] Frutos P, Hervás G, Giráldez FJ, Mantecón AR. Review. Tannins and ruminant nutrition. Span J Agric Res. 2004;2: 191–202.

[31] Patra AK, Saxena J. Exploitation of dietary tannins to improve rumen metabolism and ruminant nutrition. J Sci Food Agric. 2011;91: 24–37. doi: 10.1002/jsfa.4152 20815041

[32] Min BR, Barry TN, Attwood GT, McNabb WC. The effect of condensed tannins on the nutrition and health of ruminants fed fresh temperate forages: a review. Anim Feed Sci Technol. 2003;106: 3–19.

[33] Jeronimo E, Pinheiro C, Lamy E, Dentinho MT, Sales-Baptista E, Lopes O, et al. Tannins in ruminant nutrition: impact on animal performance and quality of edible products. In: Tannins: Biochemistry, Food Sources and Nutritional Properties. Ed. C.A. Combs. 2016;121–168.

[34] Jones WT, Mangan JL. Complexes of the condensed tannins of sainfoin (Onobrychis viciifolia Scop.) with fraction 1 leaf protein and with submaxillary mucoprotein, and their reversal by polyethylene glycol and pH. J Sci Food Agric. 1977;28: 126–136.

[35] Bhat TK, Singh B, Sharma OP. Microbial degradation of tannins–a current perspective. Biodegradation. 1998;9: 343–357. doi: 10.1023/a:1008397506963 10192896

[36] Vargas-Magaña JJ, Aguilar-Caballero AJ, Torres-Acosta JF, Sandoval-Castro CA, Hoste H, Capetillo-Leal CM. Tropical tannin-rich fodder intake modifies saliva-binding capacity in growing sheep. Animal. 2013;7: 1921–1924. doi: 10.1017/S1751731113001651 24093808

[37] Pellikaan WF, Stringano E, Leenaars J, Bongers DJ, van Laar-van Schuppen S, Plant J, et al. Evaluating effects of tannins on extent and rate of in vitro gas and CH4 production using an automated pressure evaluation system (APES). Anim Feed Sci Technol. 2011;166: 377–390.

[38] Deschamps AM, Otuk G, Lebeault J-M. Production of tannase and degradation of chestnut tannin by bacteria. J Ferment Technol. 1983;61: 55–59.

[39] Deschamps AM, Lebeault J-M. Production of gallic acid from tara tannin by bacterial strains. Biotechnol Lett. 1984;6: 237–42.

[40] Shingfield KJ, Bernard L, Leroux C, Chilliard Y. Role of trans fatty acids in the nutritional regulation of mammary lipogenesis in ruminants. Animal. 2010;4: 1140–1166. doi: 10.1017/S1751731110000510 22444614

[41] Piperova LS, Sampugna J, Teter BB, Kalscheur KF, Yurawecz MP, Ku Y, et al. Duodenal and milk trans octadecenoic acid and conjugated linoleic acid (CLA) isomers indicate that postabsorptive synthesis is the predominant source of cis-9-containing CLA in lactating dairy cows. J Nutr. 2002;132: 1235–1241. doi: 10.1093/jn/132.6.1235 12042439

[42] Natalello A, Hervás G, Toral PG, Luciano G, Valenti B, Mendoza AG, et al. Bioactive compounds from pomegranate by-products increase the in vitro ruminal accumulation of potentially health promoting fatty acids. Anim Feed Sci Technol. 2020;259: 114355.

[43] Willems H, Kreuzer M, Leiber F. Alpha-linolenic and linoleic acid in meat and adipose tissue of grazing lambs differ among alpine pasture types with contrasting plant species and phenolic compound composition. Small Rum Res. 2014;116: 153–164.

[44] Campidonico L, Toral PG, Priolo A, Luciano G, Valenti B, Hervás G, et al. Fatty acid composition of ruminal digesta and longissimus muscle from lambs fed silage mixtures including red clover, sainfoin, and timothy. J Anim Sci. 2016;94: 1550–1560. doi: 10.2527/jas.2015-9922 27136014

[45] Whitney TR, Lupton CJ, Smith SB. Redberry juniper as a roughage source in lamb feedlot rations: Wool and carcass characteristics, meat fatty acid profiles, and sensory panel traits. Meat Sci. 2011;89: 160–165. doi: 10.1016/j.meatsci.2011.04.010 21570776

[46] Whitney TR, Smith SB. Substituting redberry juniper for oat hay in lamb feedlot diets: Carcass characteristics, adipose tissue fatty acid composition, and sensory panel traits. Meat Sci. 2015;104: 1–7. doi: 10.1016/j.meatsci.2015.01.010 25678414

[47] Buccioni A, Pallara G, Pastorelli R, Bellini L, Cappucci A, Mannelli F, et al. Effect of dietary chestnut or quebracho tannin supplementation on microbial community and fatty acid profile in the rumen of dairy ewes. BioMed Res Int 2017;2017: 4969076. doi: 10.1155/2017/4969076 29457028 PMC5804114

[48] Bessa RJ, Alves SP, Jerónimo E, Alfaia CM, Prates JA, Santos‐Silva J. Effect of lipid supplements on ruminal biohydrogenation intermediates and muscle fatty acids in lambs. Eur J Lipid Sci Tech. 2007;109: 868–878.

[49] Aldai N, de Renobales M, Barron LJR, Kramer JKG. What are the trans fatty acids issues in foods after discontinuation of industrially produced trans fats? Ruminant products, vegetable oils, and synthetic supplements. Eur J Lipid Sci Tech. 2013;115: 1378–1401.

[50] Huws SA, Kim EJ, Cameron SJ, Girdwood SE, Davies L, Tweed J, et al. Characterization of the rumen lipidome and microbiome of steers fed a diet supplemented with flax and echium oil. Microbial biotech. 2015;8: 331–341. doi: 10.1111/1751-7915.12164 25223749 PMC4353346

[51] Fievez V, Colman E, Castro-Montoya JM, Stefanov I, Vlaeminck B. Milk odd- and branched-chain fatty acids as biomarkers of rumen function-an update. Anim Feed Sci Technol. 2012;172: 51–65.

[52] Vlaeminck B, Fievez V, Cabrita ARJ, Fonseca AJM, Dewhurst RJ. Factors affecting odd- and branched-chain fatty acids in milk: A review. Anim Feed Sci Technol. 2006;131: 389–417.

[53] Dias LG, Correia DM, Sá-Morais J, Sousa F, Pires JM, Peres AM. Raw bovine meat fatty acids profile as an origin discriminator. Food Chem. 2008;109: 840–847. doi: 10.1016/j.foodchem.2008.01.008 26049999

[54] Garcia PT, Pensel NA, Sancho AM, Latimori NJ, Kloster AM, Amigone MA, et al. Beef lipids in relation to animal breed and nutrition in Argentina. Meat Sci. 2008;79: 500–508. doi: 10.1016/j.meatsci.2007.10.019 22062910

[55] Morales R, Ungerfeld EM. Use of tannins to improve fatty acids profile of meat and milk quality in ruminants: A review. Chil J Agric Res. 2015;75: 239–248.

